# Sociodemographic Predictors of Breast Reconstruction Procedure Choice: Analysis of the Mastectomy Reconstruction Outcomes Consortium Study Cohort

**DOI:** 10.1155/2015/150856

**Published:** 2015-10-29

**Authors:** Tiffany N. S. Ballard, Yeonil Kim, Wess A. Cohen, Jennifer B. Hamill, Adeyiza O. Momoh, Andrea L. Pusic, H. Myra Kim, Edwin G. Wilkins

**Affiliations:** ^1^Section of Plastic Surgery, University of Michigan, Ann Arbor, MI 48109, USA; ^2^Center for Statistical Consultation and Research, University of Michigan, Ann Arbor, MI 48109, USA; ^3^Division of Plastic and Reconstructive Surgery, Memorial Sloan-Kettering Cancer Center, New York, NY 10065, USA; ^4^Department of Biostatistics, University of Michigan, Ann Arbor, MI 48109, USA

## Abstract

*Background*. To promote patient-centered care, it is important to understand the impact of sociodemographic factors on procedure choice for women undergoing postmastectomy breast reconstruction. In this context, we analyzed the effects of these variables on the reconstructive method chosen. *Methods*. Women undergoing postmastectomy breast reconstruction were recruited for the prospective Mastectomy Reconstruction Outcomes Consortium Study. Procedure types were divided into tissue expander-implant/direct-to-implant and abdominally based flap reconstructions. Adjusted odds ratios were calculated from logistic regression. *Results*. The analysis included 2,203 women with current or previous breast cancer and 202 women undergoing prophylactic mastectomy. Compared with women <40 years old with current or previous breast cancer, those 40 to 59 were significantly more likely to undergo an abdominally based flap. Women working or attending school full-time were more likely to receive an autologous procedure than those working part-time or volunteering. Women undergoing prophylactic mastectomy who were ≥50 years were more likely to undergo an abdominal flap compared to those <40. *Conclusions*. Our results indicate that sociodemographic factors affect the reconstructive procedure received. As we move forward into a new era of patient-centered care, providing tailored treatment options to reconstruction patients will likely lead to higher satisfaction and better outcomes for those we serve.

## 1. Introduction

Breast cancer, with an estimated 296,980 cases of invasive or in situ breast cancer diagnosed in 2013, is the most common non-skin cancer neoplasm of women in the US [1]. It is a diagnosis that is not limited by race, income, education, or geographic location. While many women still choose breast conservation for the primary treatment of breast cancer, the rates of mastectomy among women eligible for breast conserving surgery have increased over the past decade [2, 3]. Today most women undergo a skin-sparing mastectomy, during which the skin of the nipple, areola, and biopsy scar is removed with the breast tissue and the rest of the skin remains intact, or nipple-sparing mastectomy, in which the skin of the nipple and areola is also preserved. For women undergoing mastectomy, the benefits of breast reconstruction in preserving body image, self-esteem, sexuality, and quality of life are widely recognized [4–8]. With the growing availability of implant-based and autogenous tissue options in recent years, patients now have a wide range of procedures from which to choose. However, little is known about how and why procedure choices are made by patients and their surgeons. In fact, the decision-making process for mastectomy reconstruction remains poorly understood.

Previous studies have focused on clinical factors, such as body mass index (BMI), disease stage, and cancer treatment, as the primary determinants of procedure choice [9, 10]. However, the reconstruction decision may also be impacted by other, less obvious influences. A growing body of research suggests that sociodemographic factors have significant effects on health care treatment choices: that is, a patient's age, ethnicity, education, and income may play important roles in determining the treatment she receives [11–13]. Although the impact of these variables on the utilization of breast reconstruction has been studied in large database analyses with a focus on clinical factors [14, 15], little is known about the effects of sociodemographic variables on procedure choice in this population.

Surgeons accustomed to basing their reconstructive recommendations on purely clinical considerations may question the importance of these less “tangible” variables. However, patients' ethnic, social, educational, and financial backgrounds may significantly affect decision-making, processes of care, and outcomes [11, 16]. Growing awareness among providers and policy-makers of the importance of these factors has given rise to a “patient-centered care” movement in US health care. The concept of patient-centered care as outlined in the Institute of Medicine's (IOM) 2001 report,* Crossing the Quality Chasm*, is rooted in the recognition of patients as unique individuals [17]. The IOM calls for physicians to provide “care that is respectful and responsive to individual patient preferences, needs, and values.” Patient-centered care means tailoring treatment interventions based on patients' backgrounds and preferences. This approach rejects the traditional “one size fits all” model of health care in favor of providing individualized care, including educating patients on treatment choices and helping patients choose options which best fit their values and lifestyles.

One of the first steps in designing individualized care for breast reconstruction patients is to better understand how social and demographic factors are related to the selection of reconstructive options. In this context, we sought to assess the impact of these variables on procedure type for postmastectomy reconstruction in a large, multicenter, prospective study.

## 2. Methods

### 2.1. Study Population

Patients were recruited as part of the Mastectomy Reconstruction Outcomes Consortium (MROC) Study, a prospective, multicenter cohort study funded by the National Cancer Institute. Women 18 years or older undergoing first-time, immediate, or delayed breast reconstruction following skin-sparing or nipple-sparing mastectomy for cancer treatment or prophylaxis were eligible for participation. Immediate reconstruction was performed at the time of mastectomy, and delayed reconstruction was performed at a subsequent date. Women undergoing a secondary attempt at reconstruction or revisions were excluded. Women receiving expander/implant or abdominally based autologous tissue reconstruction were included in this analysis. Autogenous tissue reconstructions included pedicle transverse rectus abdominis musculocutaneous (pTRAM), free transverse rectus abdominis musculocutaneous (fTRAM), deep inferior epigastric perforator (DIEP), and superficial inferior epigastric artery (SIEA) flaps. Both unilateral and bilateral procedures were evaluated. Choices of reconstructive options were based on patient and surgeon preferences. Over 60 plastic surgeons from 10 centers in Michigan, New York, Illinois, Ohio, Massachusetts, Washington, DC, Georgia, Texas, and Manitoba have contributed patients to the study. The MROC Study follows patients from immediately prior to reconstruction to two years postoperatively. Appropriate approval from all participating site Institutional Review Boards (IRBs) was obtained. The electronic medical record for each patient was reviewed to obtain clinical data. All data were collected via Velos (Velos Inc., Fremont, CA), a web-based clinical trial management system.

### 2.2. Dependent Variable

In this analysis, the dependent variable of interest was reconstructive procedure choice, categorized as tissue expander/implant or abdominally based flap reconstructions. Implant procedures included tissue expander (TE) and direct-to-implant (DTI) techniques, while abdominally based flaps included pTRAM, fTRAM, DIEP, and SIEA flaps. Due to their small numbers, patients undergoing latissimus dorsi flaps (with or without implants), superior gluteal artery perforator (SGAP) flaps, and inferior gluteal artery perforator (IGAP) flaps or patients undergoing a combination of implant and autologous procedures in bilateral reconstructions were excluded.

### 2.3. Independent Variables

Demographic variables were self-reported and included age, race, ethnicity (Hispanic/non-Hispanic), marital status, employment status, smoking status, highest level of education, and household income. Categories for racial group were “white,” “black,” and “other” (American Indians, Asians, Hawaiians, and Pacific Islanders). Employment was described as full-time (including students); part-time (including those seeking employment, homemaker, or other); unable to work or disabled; or retired. The educational categories were defined as high school diploma or less; some college or college/trade degree; and some graduate school or graduate degree. Household income (dollars per year) categories included low income (<$50,000), mid income ($50,000 to $100,000), and high income (≥$100,000).

To control for potential confounding, a group of clinical variables were also incorporated into the analysis. These included body mass index (BMI), medical comorbidities, procedure laterality (unilateral versus bilateral), and cancer status. In accordance with Centers for Disease Control guidelines, BMI (kg/m^2^) was categorized as normal (less than 25), overweight (25 to less than 30), moderately obese/obesity class I (30 to less than 35), and severely obese/obesity class II (35 or above). Medical comorbidities were scored by the Charlson Index [18] and categorized as none, one, or two or more. Patients were categorized into three groups based on the indication for mastectomy and timing of the procedure: (1) patients with active cancer undergoing immediate reconstruction, (2) a history of breast cancer undergoing immediate reconstruction, or (3) a history of breast cancer undergoing delayed reconstruction. Patients with active cancer in one breast undergoing bilateral mastectomies (with a contralateral prophylactic mastectomy) and reconstruction were classified in the group of patients with active cancer undergoing immediate reconstruction. Women undergoing prophylactic mastectomies were analyzed as a separate cohort and were classified as unilateral or bilateral immediate reconstructions.

### 2.4. Statistical Analysis

As the determinants for procedural choices were expected to differ between women undergoing reconstruction following mastectomies for breast cancer and those choosing prophylactic mastectomies with reconstruction, all analyses were conducted separately for the cohorts of patients with cancer and those without cancer. Determinants for the two procedure types (abdominally based autologous flap procedures versus TE/DTI) were evaluated. Initially, chi-square tests were performed to determine significant differences in the demographic and clinical variables between procedure types. To further evaluate the effects of the sociodemographic and clinical factors on the choice of procedure, a mixed-effects logistic regression was used, with procedure type as the dependent variable and patient factors as the independent variables. The model included study site as random intercepts to adjust for between-site differences. Both unadjusted (crude) and adjusted odds ratios (OR) of choosing an abdominally based flap versus TE/DTI, along with the corresponding 95% confidence intervals, were calculated from the mixed-effects logistic regression model parameter estimates. Because the main objective of the analysis was to show the adjusted relationship between each carefully defined factor level and the choice of procedure type, even when no significant effects were seen for a factor or a subset of factor levels, all predictors were included in the model for the cohort of women undergoing reconstruction following mastectomies for breast cancer. Factors were dropped from the model for women undergoing prophylactic mastectomies if they caused quasi- or complete-separation from the small sample size. Statistical analysis was performed using Stata 13.1 (StataCorp LP, College Station, TX), and statistical significance was set at 0.05.

## 3. Results

A total of 2,405 women were included in this analysis: 2,203 women with current or previous breast cancer and 202 women undergoing prophylactic mastectomy. Demographic and clinical variables with their associated bivariate analyses are summarized in Table 1 for each cohort. Among the 2,203 women with breast cancer, 70.7% underwent TE/DTI and 29.3% received abdominally based flap reconstructions. Over 90% (*n* = 2027) underwent immediate reconstruction, while 8% (*n* = 176) underwent delayed reconstruction. For the 202 women undergoing prophylactic mastectomy and reconstruction, 77.7% received TE/DTI, while 22.3% underwent abdominally based flap reconstructions. All women undergoing prophylactic mastectomy underwent immediate reconstruction. The majority of women were white and non-Hispanic. Among all women reporting their smoking status (*n* = 2,381), 59 (2.5%) were current smokers.

### 3.1. Women with Current or Previous Breast Cancer

Bivariate analyses showed no significant differences in race, ethnicity, or marital status between women receiving TE/DTI and abdominal flaps. Reconstruction with an abdominally based flap was significantly associated with older age (*p* < 0.001); being retired or unable to work (*p* < 0.001); having a high school diploma or less (*p* < 0.001); and an annual income of less than $50,000 (*p* < 0.001). Clinical variables, including BMI, number of comorbidities, procedure laterality, cancer status, and timing of the reconstruction, were also significantly different between the two procedure types.

Table 2 summarizes the crude and the adjusted odds ratios for each factor based on the logistic regression models. Controlling for other variables in the model, age had a statistically significant effect on procedure choice: compared with women under 40 years old, those 40 to 49 and 50 to 59 years old were significantly more likely to undergo an abdominal flap than a TE/DTI reconstruction (OR 1.88, *p* = 0.009; OR 1.98, *p* = 0.006, resp.). Employment status also had a significant effect on procedure type: women working or attending school full-time were more likely to receive autologous procedures than those in part-time or volunteer occupations (OR 1.56, *p* = 0.006).

After adjusting for other variables, significant procedure choice differences were no longer observed for the education and income variables. Although not significant, statistical trends were noted for the effects of race and ethnicity on procedure choice: compared to white women, black women were less likely to undergo abdominally based flaps than TE/DTI procedures (OR 0.74, *p* = 0.25). Hispanic women were more likely to receive abdominal flaps than non-Hispanic women (OR 1.41, *p* = 0.23).

Clinical factors including BMI, laterality, and timing of reconstruction had statistically significant effects on procedure choice among women with breast cancer. Compared to women with BMIs less than 25, women with higher BMIs were more likely to undergo abdominally based flaps. Women undergoing bilateral procedures were half as likely to receive abdominal flaps compared with those undergoing unilateral reconstructions (OR 0.55, *p* < 0.001). Patients undergoing delayed reconstruction were more likely to undergo autologous procedures than their counterparts receiving immediate reconstructions (OR 11.40, *p* < 0.001).

### 3.2. Women with No Previous History of Breast Cancer

Bivariate analyses showed no significant differences in procedure choice by race, ethnicity, marital status, or employment status (Table 1). A significantly higher proportion of women undergoing prophylactic mastectomy and abdominally based flap reconstruction attended some college or had a college degree (*p* = 0.01), had an income between $50,000 and $99,999, and had BMIs over 25 (*p* < 0.001) than patients receiving TE/DTI procedures.

After adjusting for other variables, age remained significantly associated with procedure type: women with no cancer history, aged 50 years or older, were more likely to undergo an abdominal flap compared to those under 40 years old (OR 4.44, *p* = 0.02, Table 3). Race and ethnicity did not have significant effects on procedure choice for these women. Among the clinical variables, BMI had a significant effect: women with BMIs of 25 to 29.9, 30 to 34.9, and 35 and above were more likely to undergo flaps than women with BMIs less than 25 (OR 10.35, *p* = 0.001; OR 27.29, *p* < 0.001; OR 26.48, *p* < 0.001, resp.).

## 4. Discussion

Women undergoing postmastectomy reconstruction have a wide variety of reconstructive options, including tissue expander/implant-based techniques and autogenous tissue procedures utilizing a variety of donor sites. Providing patient-centered care requires awareness of patients' backgrounds and preferences in order to tailor treatment and determine the best option for each woman. Our analysis found that a number of sociodemographic variables, including age, race, ethnicity, and employment status, impact the type of reconstructive procedure received. Additional research is needed to determine whether the procedure differences observed reflect patient preferences or other more concerning factors, such as disparities either in access to care or in the quality of information received during the surgical decision-making process.

This study has multiple strengths. First, the multicenter design enables the study team to enroll patients from 10 different sites across the US and Canada, which allows us to control for site differences and increases the potential generalizability of results, compared with single-center designs. A previous study by Katz et al. demonstrated the impact that treatment site and surgeon can have on the receipt of reconstruction [19], with much wider variation in treatment rates across surgeons noted for reconstructive procedures compared to mastectomy. Therefore, controlling for site variation is a critical step in the analysis of procedure choice. The second strength is that the study is prospective, which enables us to control for multiple variables which may not be available in retrospective study designs or databases. Over 2,400 women are currently enrolled in the MROC Study and will be followed for two years postoperatively as we track longitudinal outcomes.

We found that reconstructive procedure choice varied across several demographic variables. Age plays a key role in determining which reconstructive procedure a woman chooses. Women 40 to 60 years old with current or previous cancer and women aged 50 to 60 undergoing prophylactic mastectomy were more likely to undergo an abdominally based flap compared to women less than 40 years old, a finding that has been previously demonstrated [20, 21]. A number of factors may contribute to this difference, including pregnancy, breast shape, and lifestyle. Younger women have a greater likelihood for being nulliparous with insufficient soft tissue for autologous reconstruction. Additionally, the shape of implants more closely resembles that of a nonptotic, youthful breast. Lifestyle and personal preference may also lead to younger patients' increased reluctance to undergo autologous reconstruction due to concerns over the longer recovery time and the possible impact on an active lifestyle.

Although we noted race and ethnicity effects on procedure choice, these differences were not statistically significant. In the current study, black women were less likely to undergo an abdominally based flap than white women, and Hispanic women were more likely to undergo an abdominal flap compared to non-Hispanic women. This lack of statistical significance is likely attributable to the relatively small number of minority patients in the analysis. Although the MROC Study includes 10 sites serving demographically diverse patient populations, our study cohorts are limited to evaluating the women treated at these centers. When choosing sites for MROC, particular emphasis was placed on recruiting centers from urban areas with large minority populations. However, attracting sufficient numbers of minorities for study participation remains challenging. This issue is not unique to MROC and has been described in previous prospective cohort studies and clinical trials [22, 23].

Previous large studies of databases, such as the American College of Surgeons National Surgical Quality Improvement Program (ACS-NSQIP) and the Surveillance, Epidemiology, and End Results (SEER) program, have suggested that black women are more likely to undergo autologous reconstruction compared to TE/DTI procedures [15, 21]. This observation has been attributed to minority patients' concerns over implant-related health risks and fears that reconstruction may negatively impact their cancer treatment [24, 25]. By contrast, black women in our study were less likely to undergo abdominally based flaps compared with white women. There are several possible explanations for this finding. First, the increasing use of implants nationally [26] in recent years may reflect diminishing public concerns about possible implant-related connective-tissue diseases and breast cancer. These issues prompted a Federal moratorium in April 1992, limiting implant use to reconstructive cases [27]. The moratorium was lifted in 2006 after multiple studies failed to find a link between silicone implants and systemic disease [28].

In our study, employment and education status were significant determinants of procedure choice. Women with current or previous cancer who were full-time students or employees were more likely to undergo flap-based reconstruction than TE/DTI. Even for a woman who would be a good candidate for DTI reconstruction, her surgeon cannot guarantee that an implant can be placed until examination of the mastectomy flaps intraoperatively. Therefore, women opting for implant-based reconstruction must be prepared for the possibility of requiring multiple follow-up visits for tissue expansion and a second surgery for implant exchange. These follow-up visits and subsequent surgeries requiring additional time off may negatively impact women's ability to resume working full-time. Thus, although the immediate postoperative physical recovery and restrictions are greater, autologous reconstructions may be preferred for some women as their expected return to work will be more predictable barring any major complications.

This study has several notable limitations. As with any prospective cohort design, there is potential for confounding by additional known or unknown variables. Although use of a randomized, controlled design would limit confounding, randomization would defeat the objective of this analysis, since its purpose was to examine procedure choice. Also, the feasibility of a RCT design in breast reconstruction studies is problematic, given patients' and surgeons' strong preferences for procedure types. In this study, chemotherapy and radiation were not included in the regression; these variables will be the focus of future studies assessing one- and two-year outcomes. Radiation is known to impact the reconstructive options available to a woman and has been associated with increased complications [29–32]. As mentioned above, our analyses were also limited by relatively small numbers of minority patients, despite inclusion of study sites for MROC with relatively large minority populations. While the MROC Study is a multicenter project, the majority of sites are based within large academic medical centers. Thus, our findings may not be generalizable to all patients, particularly those in smaller practice settings. Finally, we did not evaluate the mechanics of the patient decision-making process, evaluating how patients learn about their choices and subsequently choose their procedure; this is a fertile area for future research.

## 5. Conclusions

In summary, our analysis suggests that sociodemographic variables impact procedure choice in women undergoing postmastectomy breast reconstruction. Given the changing trends in mastectomy and reconstruction over the last 15 years, it is important to better understand the patient factors that impact surgical decision-making in socially, ethnically, and economically diverse populations. As we move forward into a new era of patient-centered care, providing tailored treatment options to reconstruction patients will likely lead to higher satisfaction and better outcomes for those we serve.

## Figures and Tables

**Table 1:** Patient characteristics by cancer status and procedure type.

Variable	* *Number of women (%) with current or previous cancer by reconstructive procedure type *N* = 2203			* *Number of women (%) undergoing prophylactic mastectomy by reconstructive procedure type *N* = 202		
	TE/DTI	Abdominal flap^†^	*p*	TE/DTI	Abdominal flap^†^	*p*
	1557 (70.7)	646 (29.3)		157 (77.7)	45 (22.3)	
Age (years)	1557	646	<0.001	157	45	0.01
<40	282 (84.7)	51 (15.3)		71 (85.5)	12 (14.5)	
40–49	577 (72.9)	215 (27.1)		52 (78.8)	14 (21.2)	
50–59	439 (63.8)	249 (36.2)		34 (64.2)	19 (35.8)	
≥60	259 (66.4)	131 (33.6)				
Race	1538	635	0.49	157	45	0.30
White	1346 (70.4)	565 (29.6)		147 (77.8)	42 (22.2)	
Black	114 (75.0)	38 (25.0)		8 (88.9)	1 (11.1)	
Other^*∗*^	78 (70.9)	32 (29.1)		2 (50.0)	2 (50.0)	
Ethnicity	1524	634	0.34	156	43	0.93
Non-Hispanic	1423 (70.4)	599 (29.6)		152 (78.4)	42 (21.7)	
Hispanic	101 (74.3)	35 (25.7)		4 (80.0)	1 (20.0)	
Marital status	1542	644	0.13	157	45	0.32
Not married	402 (68.1)	188 (31.9)		43 (82.7)	9 (17.3)	
Married	1140 (71.4)	456 (28.6)		114 (76.0)	36 (24.0)	
Employment status	1538	638	<0.001	155	45	0.40
Full-time/student	866 (69.3)	383 (30.7)		96 (77.4)	28 (22.6)	
Part-time/volunteer^‡^	512 (76.2)	160 (23.8)		50 (74.6)	17 (25.4)	
Unable to work/disabled	41 (61.2)	26 (38.8)		5 (100)	0 (0.0)	
Retired	119 (63.3)	69 (36.7)		4 (100)	0 (0.0)	
Smoking status	1539	643	<0.001	154	45	0.25
Never smoker	1019 (73.6)	366 (26.4)		116 (79.5)	30 (20.5)	
Previous smoker	483 (65.2)	258 (34.8)		35 (70.0)	15 (30.0)	
Current smoker	37 (66.1)	19 (33.9)		3 (100)	0 (0.0)	
Education	1549	643	<0.001	157	45	0.01
High school diploma or less	115 (51.1)	110 (48.9)		8 (88.9)	1 (11.1)	
Some college or college degree	837 (68.6)	383 (31.4)		78 (70.3)	33 (29.7)	
Some graduate or graduate degree	597 (79.9)	150 (20.1)		71 (86.6)	11 (13.4)	
Income	1505	627	<0.001	153	45	0.04
<$50,000	238 (58.5)	169 (41.5)		18 (78.3)	5 (21.7)	
$50,000–$99,999	439 (64.2)	245 (35.8)		47 (67.1)	23 (32.9)	
≥$100,000	828 (79.5)	213 (20.5)		88 (83.8)	17 (16.2)	
BMI	1557	646	<0.001	157	45	<0.001
<25	788 (84.4)	146 (15.6)		101 (93.5)	7 (6.5)	
25–29.9	458 (66.4)	232 (33.6)		35 (72.9)	13 (27.1)	
30–34.9	192 (53.3)	168 (46.7)		13 (43.3)	17 (56.7)	
≥35	119 (54.3)	100 (45.7)		8 (50.0)	8 (50.0)	
Medical comorbidities	1557	646	0.001	157	45	0.75
None	32 (64.0)	18 (36.0)		126 (78.8)	34 (21.2)	
One	1393 (72.1)	540 (27.9)		29 (74.4)	10 (25.6)	
More than two	132 (60.0)	88 (40.0)		2 (66.7)	1 (33.3)	
Procedure laterality	1557	646	<0.001	157	45	0.34
Unilateral	652 (62.9)	384 (37.1)		9 (90.0)	1 (10.0)	
Bilateral^¥^	905 (77.5)	262 (22.5)		148 (77.1)	44 (22.9)	
Cancer status, timing of reconstruction	1557	646	<0.001			
Active cancer, immediate	1492 (75.2)	492 (24.8)				
Cancer history, immediate	27 (62.8)	16 (37.2)				
Cancer history, delayed	38 (21.6)	138 (78.4)				

^†^Pedicled transverse rectus abdominis myocutaneous, free transverse rectus abdominis myocutaneous, deep inferior epigastric perforator, and superficial inferior epigastric artery flaps.

^*∗*^American Indians, Asians, Hawaiians, and Pacific Islanders.

^‡^Includes homemakers and women seeking employment.

^¥^Includes contralateral prophylactic mastectomy and reconstruction.

TE/DTI, tissue expander/direct-to-implant; BMI, body mass index.

**Table 2:** Factors associated with undergoing abdominal flap versus TE/DTI among women undergoing mastectomy for current or previous breast cancer^†^.

Variable	Unadjusted OR (95% CI)	Adjusted OR (95% CI)	*p* ^††^
Age			
<40	1.0	1.0	
40–49	2.06 (1.51–2.80)	1.88 (1.17–3.02)	0.009
50–59	3.26 (2.40–4.43)	1.98 (1.22–3.22)	0.006
≥60	2.89 (2.06–4.05)	1.63 (0.92–2.90)	0.10
Race			
White	1.0	1.0	
Black	0.77 (0.53–1.12)	0.74 (0.44–1.24)	0.25
Other^*∗*^	1.05 (0.70–1.58)	1.27 (0.65–2.50)	0.49
Ethnicity			
Non-Hispanic	1.0	1.0	
Hispanic	0.83 (0.56–1.22)	1.41 (0.81–2.48)	0.23
Marital status			
Not married	1.0	1.0	
Married	0.88 (0.72–1.07)	1.05 (0.75–1.47)	0.77
Employment status			
Part-time/volunteer^‡^	1.0	1.0	
Full-time/student	1.32 (1.08–1.62)	1.56 (1.14–2.15)	0.006
Unable to work/disabled	1.75 (1.05–2.91)	0.68 (0.31–1.46)	0.32
Retired	1.77 (1.27–2.48)	1.04 (0.58–1.87)	0.89
Smoking status			
Never smoker	1.0	1.0	
Previous smoker	1.35 (0.78–2.37)	0.54 (0.24–1.24)	0.15
Current smoker			
Education			
High school diploma or less	1.0	1.0	
Some college or college degree		1.11 (0.70–1.77)	0.66
Some graduate or graduate degree		1.19 (0.71–2.01)	0.51
Income			
<$50,000	1.0	1.0	
$50,000–$99,999	0.81 (0.63–1.03)	0.94 (0.63–1.39)	0.75
≥$100,000	0.37 (0.29–0.47)	0.76 (0.50–1.18)	0.22
BMI			
<25	1.0	1.0	
25–29.9	2.94 (2.33–3.69)	3.02 (2.15–4.25)	<0.001
30–34.9	5.25 (4.04–6.82)	5.58 (3.74–8.32)	<0.001
≥35	4.99 (3.67–6.79)	4.99 (3.12–8.00)	<0.001
Medical comorbidities			
None	1.0	1.0	
One	1.13 (0.82–1.56)	1.50 (0.62–3.62)	0.37
≥Two	1.94 (1.29–2.91)	1.63 (0.61–4.33)	0.33
Procedure laterality			
Unilateral	1.0	1.0	
Bilateral^¥^	0.51 (0.43–0.61)	0.55 (0.42–0.73)	<0.001
Cancer status, timing of reconstruction			
Active cancer, immediate	1.0	1.0	
Cancer history, immediate	1.80 (0.96–3.36)	2.09 (0.88–4.99)	0.10
Cancer history, delayed	11.01 (7.58–16.00)	11.40 (7.22–18.02)	<0.001

^†^Reference group is women undergoing tissue expander- (TE-) implant or direct-to-implant (DTI).

^††^ *p* value corresponds to the adjusted odds ratios.

^*∗*^American Indians, Asians, Hawaiians, and Pacific Islanders.

^‡^Includes homemakers and women seeking employment.

^¥^Includes contralateral prophylactic mastectomy and reconstruction.

TE/DTI, tissue expander/direct-to-implant; OR, odds ratios; BMI, body mass index.

**Table 3:** Factors associated with undergoing TE/DTI versus abdominal flap among women undergoing prophylactic mastectomy and immediate reconstruction^†^.

Variable	Unadjusted OR (95% CI)	Adjusted OR (95% CI)	*p* ^††^
Age			
<40	1.0	1.0	
40–49	2.06 (1.51–2.80)	2.03 (0.62–6.64)	0.24
50–59	3.12 (2.33–4.19)	4.44 (1.30–15.22)	0.02
Race			
White	1.0	1.0	
Black	0.77 (0.53–1.12)	0.34 (0.02–5.76)	0.46
Other^*∗*^	1.05 (0.70–1.58)	10.03 (0.51–195.93)	0.13
Marital status			
Not married	1.0	1.0	
Married	0.88 (0.72–1.07)	0.70 (0.19–2.54)	0.58
Education			
High school diploma or less	1.0	1.0	
Some college or college degree	0.48 (0.36–0.64)	6.60 (0.49–89.04)	0.16
Some graduate or graduate degree	0.26 (0.19–0.35)	4.16 (0.27–62.97)	0.30
Income			
<$50,000	1.0	1.0	
$50,000–$99,999	0.81 (0.63–1.03)	1.83 (0.34–9.93)	0.49
≥$100,000	0.37 (0.29–0.47)	1.41 (0.23–8.45)	0.71
BMI			
<25	1.0	1.0	
25–29.9	2.94 (2.33–3.69)	10.35 (2.70–39.72)	0.001
30–34.9	5.25 (4.04–6.82)	27.29 (6.45–115.49)	<0.001
≥35	4.99 (3.67–6.79)	26.48 (4.92–142.49)	<0.001
Medical comorbidities			
None	1.0	1.0	
One	1.13 (0.82–1.56)	0.84 (0.24–2.90)	0.78
≥Two	1.94 (1.29–2.91)	0.61 (0.02–15.34)	0.77
Procedure laterality, timing			
Unilateral, immediate	1.0	1.0	
Bilateral, immediate	2.68 (0.33–21.70)	6.37 (0.39–104.01)	0.19

^†^Reference group is women undergoing tissue expander- (TE-) implant or direct-to-implant (DTI).

^††^ *p* value corresponds to the adjusted odds ratios.

^*∗*^American Indians, Asians, Hawaiians, and Pacific Islanders.

TE/DTI, tissue expander/direct-to-implant; OR, odds ratios; BMI, body mass index.
